# Tuning enhanced dielectric properties of (Sc^3+^–Ta^5+^) substituted TiO_2_ via insulating surface layers

**DOI:** 10.1038/s41598-024-53046-8

**Published:** 2024-01-31

**Authors:** Wattana Tuichai, Pornjuk Srepusharawoot, Supamas Danwittayakul, Prasit Thongbai

**Affiliations:** 1 Giant Dielectric and Computational Design Research Group (GD–CDR), Department of Physics, Faculty of Science, Khon Kaen University, Khon Kaen, 40002 Thailand; 2 Institute of Nanomaterials Research and Innovation for Energy (IN–RIE), Khon Kaen University, Khon Kaen, 40002 Thailand; 3 National Metal and Materials Technology Center, 114 Thailand Science Park, Paholyothin Road, Klong 1, Klong Luang, 12120 Pathumthani Thailand

**Keywords:** Materials science, Physics

## Abstract

In this study, we achieved significantly enhanced giant dielectric properties (EG-DPs) in Sc^3+^–Ta^5+^ co-doped rutile-TiO_2_ (STTO) ceramics with a low loss tangent (tanδ ≈ 0.05) and high dielectric permittivity (ε′ ≈ 2.4 × 10^4^ at 1 kHz). We focused on investigating the influence of insulating surface layers on the nonlinear electrical properties and the giant dielectric response. Our experimental observations revealed that these properties are not directly correlated with the grain size of the ceramics. Furthermore, first-principles calculations indicated the preferred formation of complex defects, specifically 2Ta diamond and 2ScV_o_ triangular-shaped complexes, within the rutile structure of STTO; however, these too showed no correlation. Consequently, the non-Ohmic properties and EG-DPs of STTO ceramics cannot be predominantly attributed to the grain boundary barrier layer capacitor model or to electron-pinned defect-dipole effects. We also found that the semiconducting grains in STTO ceramics primarily arise from Ta^5+^, while Sc_3+_ plays a crucial role in forming a highly resistive outer surface layer. Notably, a significant impact of grain boundary resistance on the nonlinear electrical properties was observed only at lower co-dopant concentrations in STTO ceramics (1 at%). The combination of low tanδ values and high ε′ in these ceramics is primarily associated with a highly resistive, thin outer-surface layer, which substantially influences their non-Ohmic characteristics.

## Introduction

Recently, there has been a growing research interest in TiO_2_-based materials due to their enhanced giant dielectric properties (EG-DPs). These properties include high dielectric permittivity (ε′ > 10^4^), low loss tangent (tanδ < 0.1), and a low temperature coefficient of ε′ at temperatures above 150 °C^1–9^. Materials with enhanced giant dielectric properties (EG-DPs) possess great potential for applications in high-energy density storage devices and ceramic capacitors^5^.

Although CaCu_3_Ti_4_O_12_ and related ACu_3_Ti_4_O_12_ ceramics are the classic giant dielectric ceramics, exhibiting ε′ values of more than 10^4^ and low tanδ < 0.05, their ε′ values are usually temperature dependent at above 100 °C^10–14^. EG-DPs cannot be obtained from this material group. Greatly enhanced ε′ ≈ 6 × 10^4^ with low tanδ values of ≈ 0.02 for rutile-TiO_2_ ceramics was accomplished by partial co-substitution of In^3+^–Nb^5+^ ions (InNbTO)^1^. The ε′ and tanδ values of the InNbTO ceramics are dependent on the co-dopant In^3+^–Nb^5+^ concentration, which continuously increases as the doping concentration was enhanced from 0.05 to 10%, while tanδ decreased. Interestingly, it was reported that ε′ of InNbTO ceramics is independent of temperature and frequency over wide ranges. The complete details of the investigations of the EG-DPs of TiO_2_ ceramics, which were co-doped by other ion pairs such as Ga^3+^–Nb^5+^, Sm^3+^–Ta^5+^, Sc^3+^–Nb^5+^, Al^3+^–Nb^5+^, Al^3+^–Ta^5+^, and Ga^3+^–Ta^5+^, have been reported^3,15–19^. These ceramics exhibited EG-DPs. It is believed that co-doped TiO_2_ ceramics are a promising material group with high potential for use in energy-storage devices with high-energy density and capacitors. Furthermore, TiO_2_-based materials are widely utilized in various applications due to their low cost, abundance, non-toxic nature, and excellent chemical stability^20–23^.

The temperature stability of ε′ (i.e., temperature coefficient, Δε′(%)) of InNbTO ceramics and other co-doped TiO_2_ ceramics may be one of most serious problems inhibiting their practical use^24^. Improvement of the Δε′(%) value is an important research issue. Another important topic that has been extensively studied is the origination of EG-DPs of TiO_2_-based materials. Complex defect dipoles inside the grains or polarization at the interfaces of grain boundaries (GBs) and resistive outer-surface layers were proposed as the leading causes of EG-DPs^1,2,15–17,25–27^. Each proposed model is reasonable from different points of view. Thus, the actual origin of the EG-DPs of all TiO_2_ ceramics remains unclear.

The search a new co-doped TiO_2_ system that exhibits EG-DPs and/or possesses attractive electrical properties is one of the most important activities to increase the available ceramic choices for use in future applications^5^. Although the giant dielectric properties (ε′ ≈ 1.9 × 10^4^–1.4 × 10^5^) of (**A**^3+^_1/2_Ta_1/2_)_0.1_Ti_0.9_O_2_ ceramic systems (**A**TTO, **A** = In, Ga, Yb, Sm, Al, Fe, Bi, Dy, Sc, or Gd) have been presented by Li et al*.*^18^, comprehensive details of experimental results and their discussion focused only on the (Al_1/2_Ta_1/2_)_*x*_Ti_1−*x*_O_2_ system with *x* = 0–0.15. EG-DPs were obtained in the (Al_1/2_Ta_1/2_)_*x*_Ti_1−*x*_O_2_ system with *x* = 0.125 (tanδ ~ 0.054 and ε′ ~ 3.76 × 10^4^ at 1 kHz). Recently, we found that the EG-DPs of the **A**TTO family were formed in Ga^3+^–Ta^5+^ co-doped TiO_2_ (GaTaTO) materials by optimizing the sintering conditions and co-dopant concentrations^17^. Besides GaTaTO and AlTaTO ceramics, the EG-DPs of various ceramics in the **A**TTO family may be achieved.

Nonlinear current density–electric field (*J*–*E*) characteristics have been widely studied in giant-dielectric oxides, especially for CaCu_3_Ti_4_O_12_ and related ACu_3_Ti_4_O_12_ ceramics, due to their attractiveness for varistor applications^28,29^. However, the giant dielectric and nonlinear *J*–*E* characteristics of co-doped TiO_2_ in the **A**TaTO family have rarely been reported^30^. The nonlinear electrical properties in polycrystalline materials typically arise from the interface between the semiconducting and insulating components^31,32^. The observation of nonlinear *J*–*E* characteristics suggests the presence of at least one type of insulating layer, which can influence the EG-DPs of these materials. The objective of this research is to explore the EG-DPs of co-doped TiO_2_ oxides, with a particular focus on their potential use in ceramic capacitors. Additionally, the study aims to investigate the impact of the introduced insulating surface layer on the EG-DPs.

It was reported that the EG-DPs of co-doped TiO_2_ ceramics were primarily influenced by multiple factors, depending on the ionic radii of the acceptor dopants used^26^. In the InNbTO system^1,2,26^, the electron-pinned defect-dipole (EPDD) was produced, attributed to the relatively larger ionic radius of In^3+^ (*r*_6_ = 80 pm) compared to Ti^4+^ (*r*_6_ = 60.5 pm)^33^. Therefore, the predominant origin of the EG-DPs in InNbTO was ascribed to the EPDD effect. Contrarily, in GaTaTO ceramics^17^, theoretical calculations have demonstrated the absence of EPDD formation. The EG-DPs in GaTaTO ceramics were explained by extrinsic effects, such as interfacial polarization at the insulating GBs and resistive outer-surface layers, as opposed to the intrinsic EPDD effect. However, the existence of resistive outer-surface layers has yet to be proved. Furthermore, theoretical studies on the formation of EPDD have only focused on In^3+^ and G^3+^ ions. The effect of an acceptor dopant with an ionic radius intermediary to these ions, such as Sc^3+^ (r_6_ = 74.5 pm), has not been theoretically investigated.

Therefore, in this study, we successfully synthesized a novel Sc^3+^–Ta^5+^ co-doped TiO_2_ system employing a conventional solid–state reaction (SSR) method. This process resulted in EG-DPs characterized by exceptionally high ε′ of ~ 2.4 × 10^4^ and low tanδ ~ 0.05 values. First-principle calculations were employed to predict the presence of EPDDs. Additionally, we measured the nonlinear J–E properties to confirm the existence of resistive outer-surface layers. Impedance spectroscopy played a key role in revealing the formation of distinct semiconducting and insulating regions. The underlying mechanisms of the EG-DPs were comprehensively elucidated through a synergy of theoretical insights and experimental findings.

## Experimental details

An SSR technique was employed to prepare (Sc_0.5_Ta_0.5_)_*x*_Ti_1-*x*_O_2_ (*x* = 0.01, 0.025, and 0.05) powders. These ceramics are referred to as the 1%STTO, 2.5%STTO, and 5%STTO ceramics, respectively. Single-doped Sc_0.025_Ti_0.975_O_2_ (2.5%STO) and Ta_0.025_Ti_0.975_O_2_ (2.5%TTO) ceramics were also synthesized via the SSR method. The starting raw oxides, purchased from Sigma–Aldrich, consisted of Sc_2_O_3_ (99.9% purity), rutile-TiO_2_ (> 99.9%), and Ta_2_O_5_ (99.99%). Details of the SSR method for preparing co-doped TiO_2_ ceramics are given elsewhere^17^. First, the starting powders were mixed using a wet-ball milling method in ethanol for 24 h. ZrO_2_ balls, each with a diameter of 2 mm, served as the grinding media. Second, the ethanol was evaporated by heating in an oven at 90 °C for 6 h. Third, the resulting dried powders were compressed into pellets at a uniaxial pressure of 250 MPa without prior calcination or the addition of a binder. Extending the findings of our previous research^17^, which demonstrated high ε′ values exceeding 5.0 × 10^3^ in TiO_2_ co-doped with 2.5% and 5.0% (Ga^3+^–Ta^5+^), the pellets were sintered at 1500 °C for a duration of 5 h. In this work, the STTO pellets were similarly sintered at 1500 °C for 5 h.

A UV–vis Raman spectrometer (Horiba Jobin–Yvon T64000), scanning electron microscope (SEM) (SEC, SNE4500M), X-ray diffraction (XRD, PANalytical, EMPYREAN), field-emission scanning electron microscopy (FE-SEM) with energy-dispersive X-ray analysis (EDS) (HITACHI SU8030, Japan), and X-ray photoelectron spectroscopy (XPS) were employed to systematically examine the sintered STTO specimens. Comprehensive details of each technique are provided in our previous published work^17^. The sintered samples were first polished and then thermally etched at 1200 °C for 30 min. To calculate the mean grain size, the following procedure was employed: First, six different diameters were measured for each grain using the relative scale bars. Next, the average size of each individual grain was determined. Finally, the overall mean grain size for the sample was calculated, based on measurements from approximately 100 grains. The nonlinear *J*–*E* properties of as-sintered specimens were tested at ~ 25 °C (Keithley Model 247). The α value was calculated using the following formula:

1 $${\upalpha } = \frac{{\log \left( {{\text{J}}_{2} /{\text{J}}_{1} } \right)}}{{\log \left( {{\text{E}}_{2} /{\text{E}}_{1} } \right)}},$$

where E_1_ and E_2_ represent the electric fields, at which J_1_ = 1 and J_2_ = 10 mA cm^−2^, respectively. E_b_ was defined as equal to being *E*_1_^31,32,34,35^. Capacitance (C_p_) and tanδ values of as-sintered specimens were determined as a function of frequency (40–10^7^ Hz) and temperature (− 60 to 210 °C) by employing a KEYSIGHT E4990A Impedance Analyzer. The ε′ value was calculated by the equation,

2 $${\upvarepsilon }^{\prime } = \frac{{{\text{C}}_{{\text{p}}} {\text{d}}}}{{{\upvarepsilon }_{0} {\text{A}}}},$$

where A and d represent the electrode area and sample thickness, respectively. ε_0_ = 8.854 × 10^–12^ F/m. The complex dielectric constant (ε^*^) and complex impedance (Z^*^) were calculated from the equations,

3 $${\upvarepsilon }^{*} = {\upvarepsilon }^{\prime } - i{\upvarepsilon }^{\prime \prime } = \left( {i\omega C_{0} Z^{*} } \right)^{ - 1} = \left[ {i\omega C_{0} \left( {{\text{Z}}^{\prime } - i{\text{Z}}^{\prime \prime } } \right)} \right]^{ - 1} ,$$

where ε′ and ε′′ represent the real and imaginary parts of ${\upvarepsilon }^{*}$ (ε′′ = ε′tanδ), while Z′ and Z′′ represent the real part and imaginary parts of Z*, respectively. ${{\text{C}}}_{0}={\upvarepsilon }_{0}{\text{A}}/{\text{d}}$ is the empty cell capacitance. The most preferred configuration for STTO ceramics was determined for the DFT calculations. Details of our computational calculations are given elsewhere^17^.

## Results and discussion

**Figure 1:**
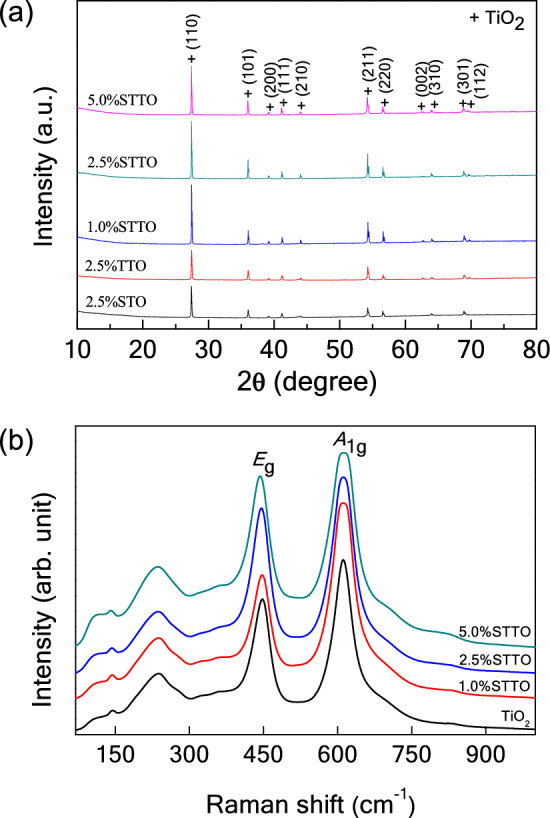
(**a**) XRD patterns of sintered 2.5%STO, 2.5%TTO, 1.0%STTO, 2.5%STTO, and 5.0%STTO ceramics. (**b**) Raman spectra of TiO_2_, 1.0%STTO, 2.5%STTO, and 5.0%STTO ceramics.

Figure 1a gives XRD patterns of single as well as co-doped specimens, confirming that the main phase of rutile-TiO_2_ (JCPDS 21-1276) contains no impurity phases. Both the ionic radii Sc^3+^ (*r*_*6*_ = 0.745 Å) and Ta^5+^ (*r*_6_ = 0.64 Å) dopants are larger than the host Ti^4+^ ion (*r*_6_ = 0.605 Å)^33^. Thus, cell parameters of the rutile-structure may be changed by doping with Sc^3+^ and/or Ta^5+^ ions. Consequently, the lattice parameters (*a* and *c* values) were obtained from Rietveld refinement method. TiO_2_, 2.5%STO, 2.5%TTO, 1%STTO, 2.5%STTO and 5%STTO ceramics showed respective *a* values of 4.593, 4.595, 4.595, 4.598, 4.598 and 4.601 Å, while *c* values were 2.959, 2.960, 2.962, 2.965, 2.965 and 2.969 Å, respectively. The unit cell volumes were 62.44, 62.50, 62.54, 62.68, and 62.68 Å, respectively. Single and co-doped ceramic *a* and *c* values are slightly greater than for a pure TiO_2_ ceramic. Therefore, both Sc^3+^ and Ta^5+^ dopant ions could be substituted into the rutile-TiO_2_ structure.

Figure 1b shows Raman spectra of STTO specimens compared to that of a pure TiO_2_ material. Overall, the Raman spectra are similar to co-doped TiO_2_ systems reported in literature^17,19,30,36,37^. Here, we focused on the strongest *E*_g_ and* A*_1g_ peaks. These affect the giant dielectric response of TiO_2_-based oxides. These two main peaks are usually associated with oxygen vacancies and O–Ti–O bonds, respectively^37^. *E*_g_ mode Raman peaks for TiO_2_, 1%STTO, 2.5%STTO and 5%STTO ceramics are, respectively, at 447.9, 447.1, 446.3, and 443.3 cm^−1^, while the *A*_1g_ peaks are at 611.3, 610.1, 611.2, and 610.2 cm^−1^, respectively. The *E*_g_ peak of the STTO specimens shifted to lower wave numbers as the Sc^3+^ and Ta^5+^ co-dopant concentrations increased from 0 to 5 at.%, whereas the *A*_1g_ peak is not changed significantly. Generally, substitution of an acceptor dopant, such as Sc^3+^, into the TiO_2_ structure requires oxygen vacancies for charge compensation, following Eq. (1). According to this equation, the nominal composition of the 5%STTO ceramic with an *E*_g_ peak appeared at 443.33 cm^−1^, the theoretical ratio of [O]/[Ti] in the 5%STTO ceramic should be 1.987. This result is in agreement with Parker et al*.*^38^. They reported that the TiO_2−*x*_* E*_g_ peak decreased from 447 to 443 cm^−1^ as the [O]/[Ti] ratio in a rutile-TiO_2_ was reduced, from 2.0 to 1.99. The *E*_g_ peak shifting to lower a wave number confirms that the presence of oxygen vacancies in STTO materials, which increased with the Sc^3+^ concentration. Oxygen vacancies detected using Raman analysis confirms the origin of the enlarged grain size of the co-doped 5.0%STTO ceramic was due to diffusion of oxygen vacancies when compared to that of the 2.5%TTO ceramic, since the average grain size of the 2.5%TTO specimen enlarged with addition of 2.5 at% Sc^3+^ ions (5.0%STTO).

The XPS technique was used to further analyze the possible effects of the dopants on the presence of defects in co-doped STTO materials. As illustrated in Fig. S1a (supplementary Information), the fitted XPS peaks of O1*s* confirmed the oxygen lattices, oxygen vacancies, and surface hydroxyl (OH) groups in the 5.0%STTO ceramic^1,16,36^. Therefore, it can be confirmed that substitution of Sc^3+^ can contribute to promoting oxygen vacancies, following Eq. (1). Furthermore, the presence of Ti^4+^ and Ti^3+^ was confirmed^1,39^, Fig. S1b. Furthermore, the XPS results also showed Ta^5+^ (Fig. S1c)^39,40^ and Sc^3+^ (Fig. S1d)^15^. The Ti^3+^/Ti^4+^ ratio of the 5.0%STTO material was found 4.84%, which was larger than the expected ratio calculated from the nominal composition of the 5.0%STTO ceramic (2.63%), following:

4 $$2{\text{TiO}}_{2} + {\text{Ta}}_{2} {\text{O}}_{5} \mathop{\longrightarrow}\limits^{{4{\text{TiO}}_{2} }}2{\text{Ti}}_{{{\text{Ti}}}}^{\prime } + 2{\text{Ta}}_{{{\text{Ti}}}}^{ \cdot } + 8{\text{O}}_{O} + 1/2{\text{O}}_{2} ,$$

5 $${\text{Ti}}^{4 + } + e \to {\text{Ti}}^{3 + } .$$

A higher Ti^3+^/Ti^4+^ ratio is generally due to the oxygen loss during sintering, following the relationship.

6 $${\text{O}}_{{\text{O}}}^{x} \to \frac{1}{2}{\text{O}}_{2} + V_{{\text{O}}}^{ \cdot \cdot } + 2e^{\prime } .$$

**Figure 2:**
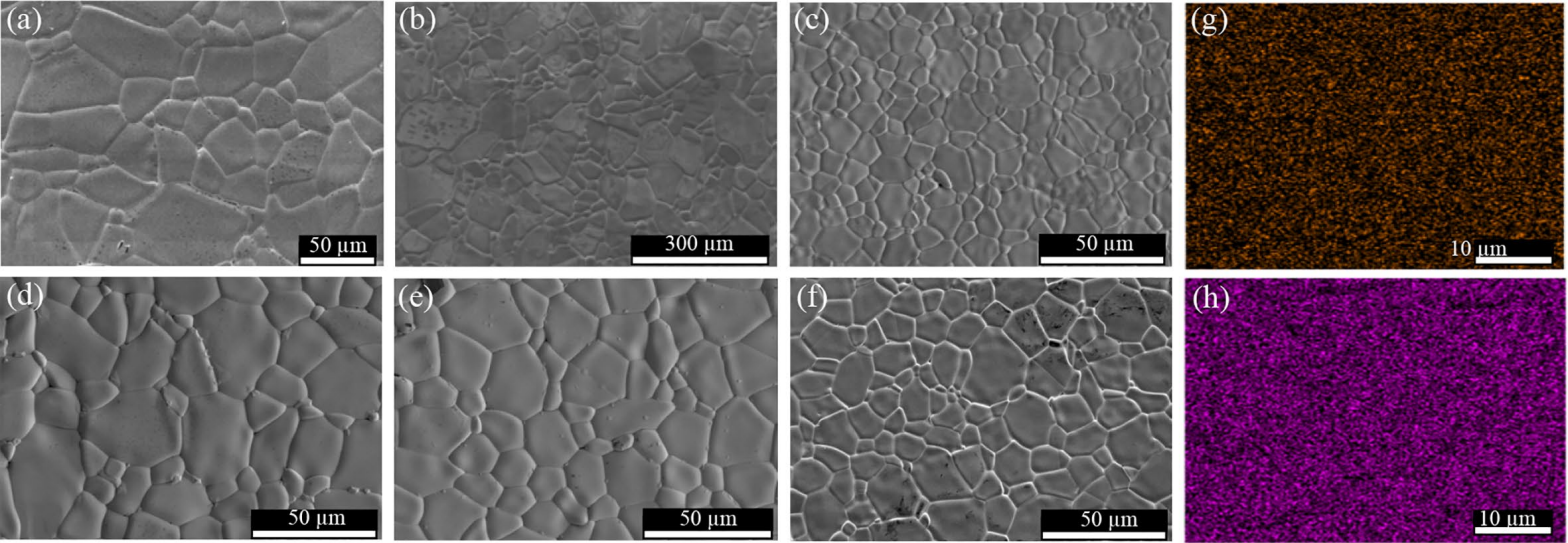
SEM images of surface morphologies of (**a**) TiO_2_, (**b**) 2.5%STO, (**c**) 2.5%TTO, (**d**) 1.0%STTO, (**e**) 2.5%STTO, and (**f**) 5.0%STTO ceramics. Elemental mapping of the 5%STTO ceramic, (**g**) Sc and (**h**) Ta.

Figure 2a–f reveal the effects of Sc^3+^ and Ta^5+^ dopants upon the microstructural evolution of TiO_2_ specimens. Highly dense materials with no porosity are achieved in these sintered materials. Average grain sizes of the un-doped TiO_2_, single-doped 2.5%TTO, and 2.5%STO are about 42.9 ± 16.0, 12.6 ± 4.1, and 85.6 ± 33.6 µm, respectively. Only doping TiO_2_ with Sc^3+^ ions causes a great increase in an average grain size, by ~ 2 times. This result is likely attributed to diffusion of oxygen vacancies ($V_{{\text{O}}}^{ \cdot \cdot }$), which are produced as part of the Sc^3+^-doped TiO_2_ structure owing to charge compensation, following:

7 $${\text{Sc}}_{2} {\text{O}}_{3} \mathop{\longrightarrow}\limits^{{2{\text{TiO}}_{2} }}2{\text{Sc}}_{{{\text{Ti}}}}^{\prime } + V_{{\text{O}}}^{ \cdot \cdot } + 3{\text{O}}_{{\text{O}}} .$$

A highly enlarged grain size of the 2.5%STO ceramic is similar to that described by Tuichai et al.^17^ for a Ga^3+^-doped TiO_2_ ceramic. Doping TiO_2_ with a pentavalent cation, such as Ta^5+^, resulted in a remarkable reduction in a grain size. The primary contribution of the Ta^5+^ dopant is to suppress the GB mobility^17^.

The roles of Sc^3+^ and Ta^5+^ ions on the microstructural evolution of TiO_2_ ceramics are totally different. The combination effect of co-doped Sc^3+^–Ta^5+^ ions was therefore studied to determine the dominant effect of Sc^3+^ or Ta^5+^. Mean grain sizes of co-doped 1%STTO, 2.5%STTO, and 5%STTO specimens are about 27.3 ± 10.5, 20.2 ± 5.1, and 17.8 ± 6.9 µm, respectively. Although these grain sizes are between those of the 2.5%STO and 2.5%TTO specimens, they are closer to a single-doped 2.5%TTO material than that of the 2.5%STO ceramic. These indicate that the restorative force inhibiting GB migration caused by the Ta^5+^ dopant is more dominant than that of the driving force for promoting grain growth that primarily resulted from the Sc^3+^ dopant^17^.

Dopant dispersion in STTO ceramics is revealed in the elemental images shown in Fig. 2g, h. The Ta and Sc dopants are observed to homogeneously disperse throughout the microstructure with no segregation towards any specific region.

**Figure 3:**
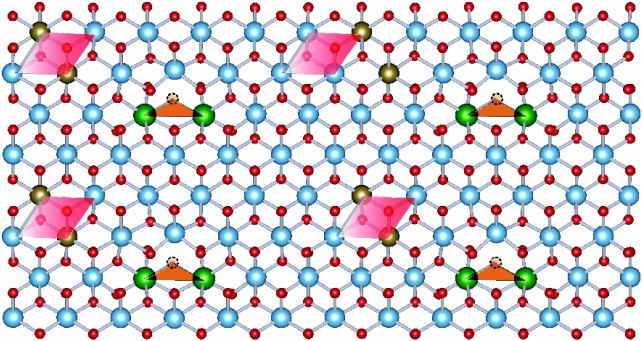
The lowest energy configurations of (Ti–2Sc–V_o_)O_2_ triangular shaped complex, (Ti–2Ta)O_2_ diamond shaped defect, and triangular and diamond shaped defects of Sc^3+^–Ta^5+^ co-doped TiO_2_ ceramics.

It was suggested that the EPDDs in co-doped TiO_2_ ceramics is determined by the ionic size of the acceptor dopant^26,37^. Ga^3+^–Nb^5+^ and Ga^3+^–Ta^5+^ co-dopants in TiO_2_ ceramics cannot create EPDDs owing to the lower ionic radius of Ga^3+^ (*r*_6_ = 62.0 pm) compared to that of an In^3+^ dopant (*r*_6_ = 80.0 pm)^33^. Considering that the ionic radius of Sc^3+^ (*r*_6_ = 74.5 pm) is between that of Ga^3+^ and In^3+^ ions^33^, EPDDs could be formed in the current study. Thus, the possible formation of EPDDs in Sc^3+^–Ta^5+^ co-doped TiO_2_ ceramics is theoretically predicted using first-principles calculations. An oxygen vacancy was shown to exist the XPS and Raman results. Thus, for the first step of the calculation, one oxygen atom was removed from the rutile structure, and two Sc atoms were substitute into the positions of two Ti atoms_._ Such a defect cluster is referred to (Ti–2Sc–V_o_)O_2_. Various characteristics of the defect cluster were tested. For each characteristic, all atoms were allowed to relax completely with no symmetrical constraints. As presented in Fig. 3, by considering the total energy, the (Ti–2Sc–V_o_)O_2_ characteristic with a triangular shape was the most stable. According to our previous work ^17^, with substitution of two Ta atoms in the rutile TiO_2_ structure, the most preferable structure of a (Ti–2Ta)O_2_ defect cluster was diamond shaped. Finally, the lowest energy configuration, which indicates the most stable among the diamond-shaped (Ti–2Ta)O_2_ and triangular–shaped (Ti–2Sc–V_o_)O_2_ defect clusters, was then was calculated. The result showed that these two types of defect clusters are preferentially separated from each other, as illustrated in Fig. 3. Thus, EPDDs are not created in Sc^3+^–Ta^5+^ co-doped TiO_2_ ceramics.

**Figure 4:**
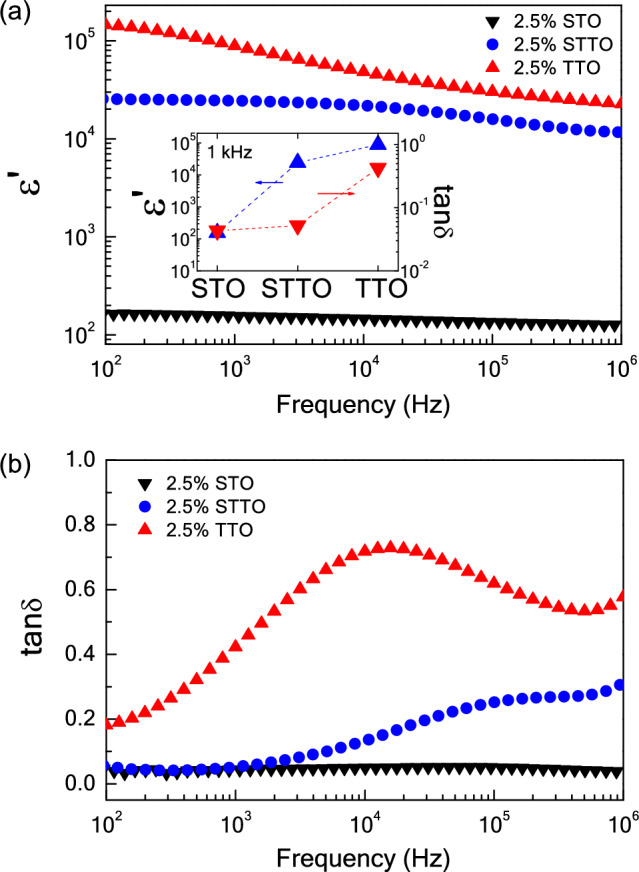
(**a**) Dielectric permittivity (ε′) as a function of frequency at 30 °C for single − doped TiO_2_ ceramics (2.5%STO and 2.5%TTO) and co–doped TiO_2_ (2.5%STTO) ceramic; inset reveals the ε′ and tanδ values (1 kHz and 30 °C). (**b**) tanδ as a function of frequency at 30 °C.

Influences of Sc^3+^ and Ta^5+^ dopants on the dielectric response in TiO_2_ materials was explored at ~ 25 °C from 10^2^ to 10^6^ Hz. As displayed in Fig. 4, the ε′ of the 2.5%TTO was very high (10^4^–10^5^), while its tanδ was also very large. Thus, EG-DPs cannot be obtained in the 2.5%TTO ceramic. This result is usually seen in Nb^5+^ and Ta^5+^ doped TiO_2_ ceramics^1,17,26^. Alternatively, both the ε′ and tanδ values of the 2.5%STO ceramic were very low, ~ 150 and 0.03, respectively. The dielectric characteristics of the single-doped 2.5%STO ceramic are similar to those reported for acceptor doped TiO_2_ ceramics, such as Al^3+^–, In^3+^–, and Ga^3+^-doped TiO_2_ ceramics^1,16,17,30^. Therefore, the EG-DPs of TiO_2_ cannot be accomplished in single-doped Ta^5+^ or Sc^3+^. Nevertheless, EG-DPs can be accomplished by co-doping with Sc^3+^–Ta^5+^. A high ε′ of 2.4 × 10^4^ with a low tanδ ~ 0.06 was obtained, as shown in the inset of Fig. 4a. According to the first principles calculations, the EG-DPs of Sc^3+^–Ta^5+^ co-doped TiO_2_ materials are not likely attributable to the EPDDs. The defect clusters associated with the Ta^5+^ and Sc^3+^ dopants are not correlated. Therefore, these EG-DPs should be attributed to extrinsic factors such the internal and/or surface barrier layer capacitor (IBLC/SBLC) effects.

**Figure 5:**
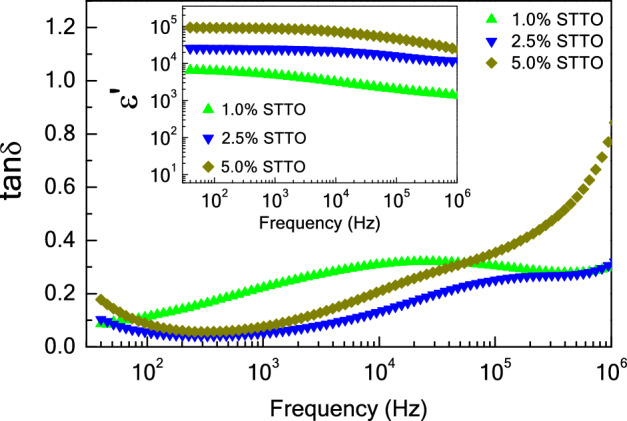
Frequency dependence of tanδ at 30 °C for Sc^3+^/Ta^5+^ co–doped TiO_2_ ceramics with different co–dopant concentrations; inset shows the frequency dependence of dielectric permittivity (ε′) at 30 °C.

To further describe the primary cause of EG-DPs, the effect of co-dopant content on the dielectric characteristics of Sc^3+^–Ta^5+^ co-doped TiO_2_ materials was further studied. As demonstrated in Fig. 5 and its inset, the ε′ of the Ta^5+^–Sc^3+^ co-doped TiO_2_ materials increases with the Ta^5+^–Sc^3+^ content from 1.0 to 5.0% over a measured frequency range. From frequencies of 10^2^–10^5^ Hz, the tanδ of the 1.0%STTO material was the largest. Values of tanδ at 1 kHz for the 1.0%SSTO, 2.5%SSTO, and 5.0%SSTO ceramics were 0.22, 0.05, and 0.07, respectively. EG-DPs of the Sc^3+^–Ta^5+^ co-doped TiO_2_ materials are similar to that found in the (Zn^2+^–Nb^5+^)^4^, (Ga^3+^–Ta^5+^)^17^, (In^3+^–Nb^5+^)^1,24,25^, (Sc^3+^–Nb^5+^)^15^, (Al^3+^–Nb^5+^)^37^, and (Ga^3+^–Nb^5+^)^26^ co-doped TiO_2_ systems. The IBLC and SBLC models^41^ indicated that the giant dielectric response is dependent on the charge carrier density inside the semiconducting portion (*semi*-P), the *C* value at the internal interface between the (*semi*-Ps) and insulating regions (*in*-Ps). Conductivity and tanδ are dependent on the resistivity of the *in*-Ps. According to Eq. (2), the free charge concentration in Sc^3+^–Ta^5+^ co-doped TiO_2_ materials is increased with the Ta^5+^ content. Thus, more charge carriers inside the *in*-Ps trapped at the internal interface of the *in*-Ps gives rise to significantly increased ε′ value.

**Figure 6:**
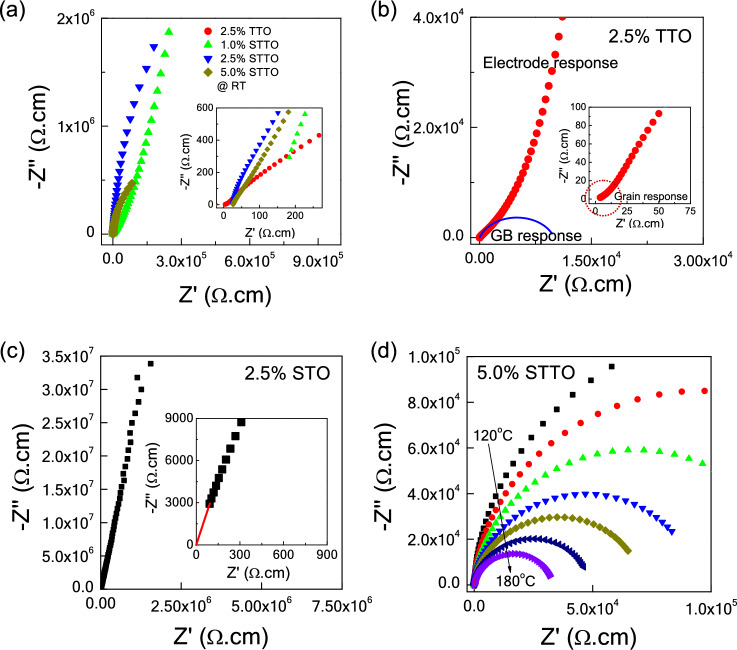
(**a**) Z* plots at RT for Ta single–doped and (Sc^3+^–Ta^5+^) co–doped TiO_2_ ceramics; inset shows an expanded view near the origin. (**b**) Z* plot of 2.5%TTO ceramics; the blue solid curve is the estimated R_gb_ value. Inset of (**b**) illustrates Z* plot close to the origin, showing the electrical response of the semiconducting grains of the 2.5%TTO ceramic. (**c**) Z* plot of 2.5%STO ceramic at RT; inset demonstrates an expanded view close to the origin, showing zero intercept on Z′ axis. (**d**) Z* plots at various temperatures for the 2.5%STTO ceramic.

Impedance spectroscopy was carried out to further confirm the presence of *semi*-Ps and *in*-Ps in Sc^3+^–Ta^5+^ co–doped TiO_2_ materials. Figure 6a and its inset show impedance complex plane (Z*) plots at ~ 25° and nonzero intercept on the Z′-axis at high-frequencies for Sc^3+^–Ta^5+^ co-doped TiO_2_ materials compared to that of Ta^5+^ single-doped TiO_2_. A full semicircular arc was not observed at ~ 25 °C for any of the samples. Only portions of a large semicircular arc are appeared in the Z* plot. The observed large arcs and nonzero intercepts in the Sc^3+^–Ta^5+^ co-doped TiO_2_ materials indicates the electrical responses of the *in*-Ps and *semi*-Ps, respectively^42^. Resistance of the *semi*-Ps significantly decreased with increasing co-dopant content, from 1.0 to 2.5%, following Eqs. (2) and (3). However, a nonzero intercept was observed in the single-doped 2.5%TTO ceramic. Clearly, a small semicircular arc is observed in this ceramic (inset of Fig. 6b) with relatively large semicircular arcs of the GB and electrode responses. Formation of *semi*-Ps (grains) in TiO_2_ is usually caused by substitution of pentavalent ions, following Eqs. (2) and (3). Alternatively, substitution of Sc^3+^ cannot create *semi*-Ps, as displayed in Fig. 6c and its inset. Only parts of a large arc are observed with no nonzero intercept. This can be explained by Eq. (1), where $V_{{\text{O}}}^{ \cdot \cdot }$ was created in the single-doped 2.5%STO ceramic, rather than free electrons. A complete large arc can be observed at high temperatures for all co-doped ceramics, as demonstrated in Fig. 6d for the 5.0%STTO material. This result indicates that the resistance of the *in*-Ps decreases with increasing temperature. According to the impedance spectroscopy, the EG-DPs of the STTO materials should primarily be attributed to extrinsic factors such as the IBLC/SBLC effect.

**Figure 7:**
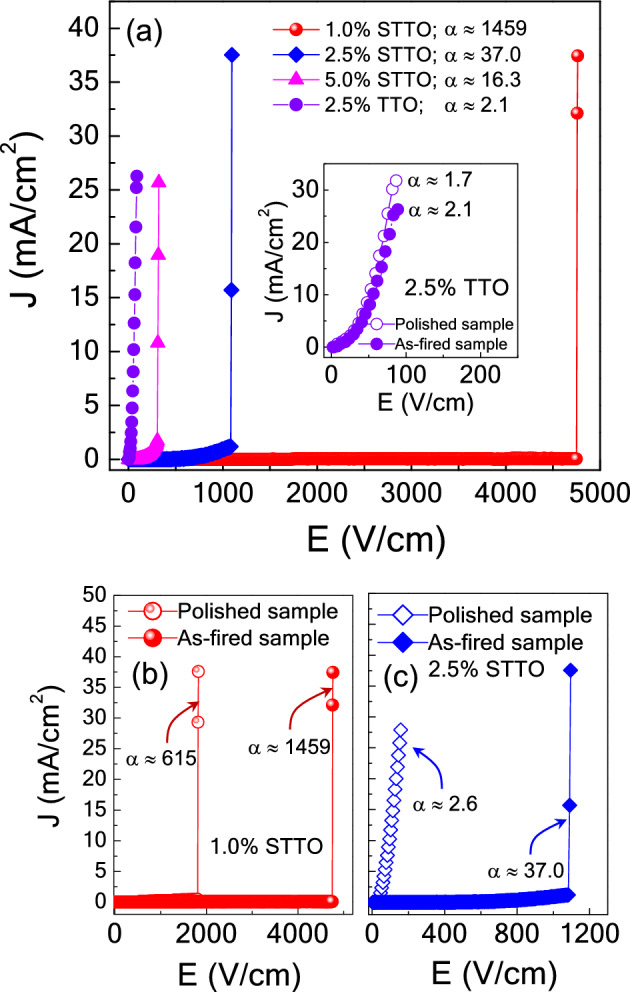
(**a**) Nonlinear *J*–*E* properties of the 2.5%TTO ceramic and all Sc^3+^/Ta^5+^ co–doped TiO_2_ ceramics at RT; inset shows *J*–*E* characteristics of the 2.5%TTO ceramic before (as-fired sample) and after polishing outer surfaces (polished sample). (**b**, **c**) *J*–*E* characteristics of the co-doped 1.0%STTO and 2.5%STTO ceramics before and after polishing the outer surface.

The nonlinear *J*–*E* characteristics of the single and co-doped TiO_2_ ceramics were investigated at ~ 25 °C. As shown in Fig. 7a, all as-sintered ceramics exhibit nonlinear *J*–E properties. Their E_b_ and α values significantly increased with decreasing co-dopant concentration. Surprisingly, the α value of the 1.0%STTO ceramic was very, ~ 1459 V/cm, calculated in the *J* range of 1–10 mA/cm^2^. The α value of the 2.5%STTO ceramic was also very large, ~ 37.0 V/cm, compared to that of CaCu_3_Ti_4_O_12_-based ceramics^13^. Nevertheless, as shown in the inset of Fig. 7a, the E_b_ and α values of the single-doped 2.5%TTO ceramic were very low (~ 2 V/cm) since there was no acceptor Sc^3+^ dopant in the sample. This result clearly shows the essential role of acceptor-Sc^3+^ dopant ions to form the *in*-Ps. It is noteworthy that both the E_b_ and α values of the 2.5%STTO and 5.0%STTO materials were lower than that of the 1.0%STTO ceramic. According to the impedance spectroscopy results, the resistance of the *semi*-Ps for the 2.5%STTO and 5.0%STTO ceramics was smaller than that of the 1.0%STTO ceramic, indicating that the free charge concentration (N_s_) in these two samples is higher than that of the 1.0%STTO ceramic. According to the double Schottky barrier models in polycrystalline ceramics^42^, the potential barrier height at the internal insulating interface (Φ_b_) is reduced with increasing N_s_ in the *semi*-Ps. The significantly decreased Φ_b_ values of the 2.5%STTO and 5.0%STTO ceramics may be the primary cause of the decreased E_b_ values, even though their GB densities were larger than that of the 1.0%STTO ceramic (due to larger average grain sizes).

According to our previous work^27^, the E_b_ value of as-fired InNbTO materials was much larger than that of polished samples. This result indicates that the outer surface layer (or SBLC) of the InNbTO materials had a remarkable impact on its J–E characteristics. Therefore, the effect of the surface layer was studied. We first examined the 2.5%TTO ceramic. These results are shown in the inset of Fig. 7a. The outer surface layer (or SBLC) has an effect on the J–E character of the single-doped 2.5%TTO ceramic. Both the E_b_ and α values of the as-fired and polished specimens are nearly the same. In other words, an SBLC was not formed on the surface of the as-fired 2.5%TTO ceramic due to the absence of an acceptor dopant. The weak nonlinear J–E properties of the 2.5%TTO ceramic result from the weak effect of the IBLC at the GBs. As illustrated in Fig. 7b and c, after removing the outer surface layer, strong nonlinear J–E properties of the polished 1.0%STTO ceramic were found, with an extremely high α value, ~ 615. However, strong nonlinear J–E properties of the polished 2.5%STTO ceramic were not obtained with a low α value, ~ 2.6. This result clearly shows that the SBLC effect was dominant in the 2.5%STTO and 5.0%STTO ceramics. Unfortunately, it should be emphasized that the nonlinear J–E characteristic of the 1.0% STTO ceramic did not exhibit reversibility following the measurement. While the nonlinear J–E characteristic of the 1.0% STTO ceramic precludes its application in varistor devices, this experiment highlighted the significant role of the outer surface layer in the EG-DPs. Therefore, the EG-DPs (high ε′ and low tanδ) of STTO ceramics are attributed to the SBLC effect. However, if we consider only a high ε′ neglecting a low tanδ, such a high ε′ is caused by the IBLC and sample-electrode interface effects. When the outer surface was removed, a high ε′ could be obtained, while tanδ was also very large. This research provides comprehensive guidance for achieving high-performance giant-dielectric response in co-doped TiO_2_ ceramics by inhibiting the formation of non-Ohmic sample-electrode contact via creation of a highly resistive outer surface layer.

## Conclusions

Excellent giant dielectric properties with very high ε′ ≈ 2.4 × 10^4^ and low tanδ ≈ 0.05 coupled with strong non-Ohmic properties with high E_b_ and α were observed in as-fired STTO ceramics. Based on microstructural analysis and first-principles calculations, these two interesting electrical properties were not primarily caused by the IBLC or EPDD effects. Systematically investigated results clearly show that free charges inside a semiconducting inner core or grain interiors of STTO ceramics were induced by Ta^5+^ dopant ions. A highly resistive outer surface layer of STTO ceramics, associated with Sc^3+^ dopant ions, was the key factor contributing to the strong non-Ohmic properties and low tanδ values. The GB contribution to the non-Ohmic properties was only observed in the STTO ceramic that was co-doped with 1%(Sc + Ta).

### Supplementary Information

Supplementary Information.

## Data Availability

The data of this study are available from the corresponding author upon reasonable request.
