# Supplementary material for: Tuning enhanced dielectric properties of (Sc3+–Ta5+) substituted TiO2 via insulating surface layers
Source: Sci Rep. 2024 Jan 31;14:2593. doi: 10.1038/s41598-024-53046-8 (PMC10830478; doi:10.1038/s41598-024-53046-8)
Supplement: Supplementary file 1 — Supplementary Information. [file 41598_2024_53046_MOESM1_ESM.doc]

Supplementary Information

Tuning enhanced dielectric properties of (Sc^3+^–Ta^5+^) substituted TiO_2_ *via* insulating surface layers

Wattana Tuichai^1^, Pornjuk Srepusharawoot^1,2^, Supamas Danwittayakul^3^, Prasit Thongbai^1,2^,*

^1^Giant Dielectric and Computational Design Research Group (GD**–**CDR), Department of Physics, Faculty of Science, Khon Kaen University, Khon Kaen 40002, Thailand

^2^Institute of Nanomaterials Research and Innovation for Energy (IN**–**RIE), Khon Kaen University, Khon Kaen 40002, Thailand

^3^National Metal and Materials Technology Center, 114 Thailand Science Park, Paholyothin Road, Klong 1, Klong Luang, Pathumthani 12120, Thailand

*E–mail address: [pthongbai@kku.ac.th](mailto:pthongbai@kku.ac.th) (P. Thongbai)

1. XRS spectra of the 5.0%STTO ceramic: (a) O 1*s*, (b) Ti 2*p*, (c) Ta 4*f*, and (d) Sc 2*p*.

As illustrated in Fig. S1 (supplementary Information), according to fitting results, the highest peak at 529.77 eV resulted from the oxygen lattice, *i.e.*, O−Ti bond, as is usually found in the XPS spectrum of an undoped TiO_2_ ceramic^1^. The fitted peaks at 531.02, 532.07, and 532.92 eV are attributed to the oxygen lattices of other bonds (O−Sc and O−Ta), oxygen vacancies, and surface hydroxyl (OH) groups, respectively^1-3^. As depicted Fig. S1b, the position of the Ti 2*p*_3/2_ at 458.52 eV shows the presence of Ti^4+ 1^. According to the fitted result, a peak at 457.68 eV confirms the Ti^3+1,4^. Furthermore, the XPS results also showed that Ta 4*f* consisted of two peaks, at 25.85 (4 *f*_7/2_) and 27.70 eV (4 *f*_5/2_). This indicates Ta *4f* has an oxidation state of Ta^5+^ ^4,5^, Fig. S1c. Also, the Sc 2*p* peaks at 401.95 and 406.4 eV signify Sc^3+6^, Fig. S1d.

**References**

1 Hu, W. *et al.* Electron-pinned defect-dipoles for high-performance colossal permittivity materials. *Nat. Mater.* **12**, 821-826, doi:10.1038/nmat3691

http://www.nature.com/nmat/journal/v12/n9/abs/nmat3691.html#supplementary-information (2013).

2 Liu, G., Fan, H., Xu, J., Liu, Z. & Zhao, Y. Colossal permittivity and impedance analysis of niobium and aluminum co-doped TiO_2_ ceramics. *RSC Advances* **6**, 48708-48714, doi:10.1039/C6RA07746C (2016).

3 Song, Y. *et al.* The contribution of doped-Al to the colossal permittivity properties of Al_x_Nb_0.03_Ti_0.97-x_O_2_ rutile ceramics. *Journal of Materials Chemistry C* **4**, 6798-6805, doi:10.1039/C6TC00742B (2016).

4 Sheppard, L. R., Holik, J., Liu, R., Macartney, S. & Wuhrer, R. Tantalum Enrichment in Tantalum-Doped Titanium Dioxide. *J. Am. Ceram. Soc.* **97**, 3793-3799, doi:10.1111/jace.13201 (2014).

5 Obata, K., Irie, H. & Hashimoto, K. Enhanced photocatalytic activities of Ta, N co-doped TiO_2_ thin films under visible light. *Chem. Phys.* **339**, 124-132, doi:<http://dx.doi.org/10.1016/j.chemphys.2007.07.044> (2007).

6 Tuichai, W., Danwittayakul, S., Chanlek, N., Thongbai, P. & Maensiri, S. High-performance giant-dielectric properties of rutile TiO_2_ co-doped with acceptor-Sc^3+^ and donor-Nb^5+^ ions. *J. Alloys Compd.* **703**, 139-147, doi:<http://dx.doi.org/10.1016/j.jallcom.2017.01.333> (2017).
